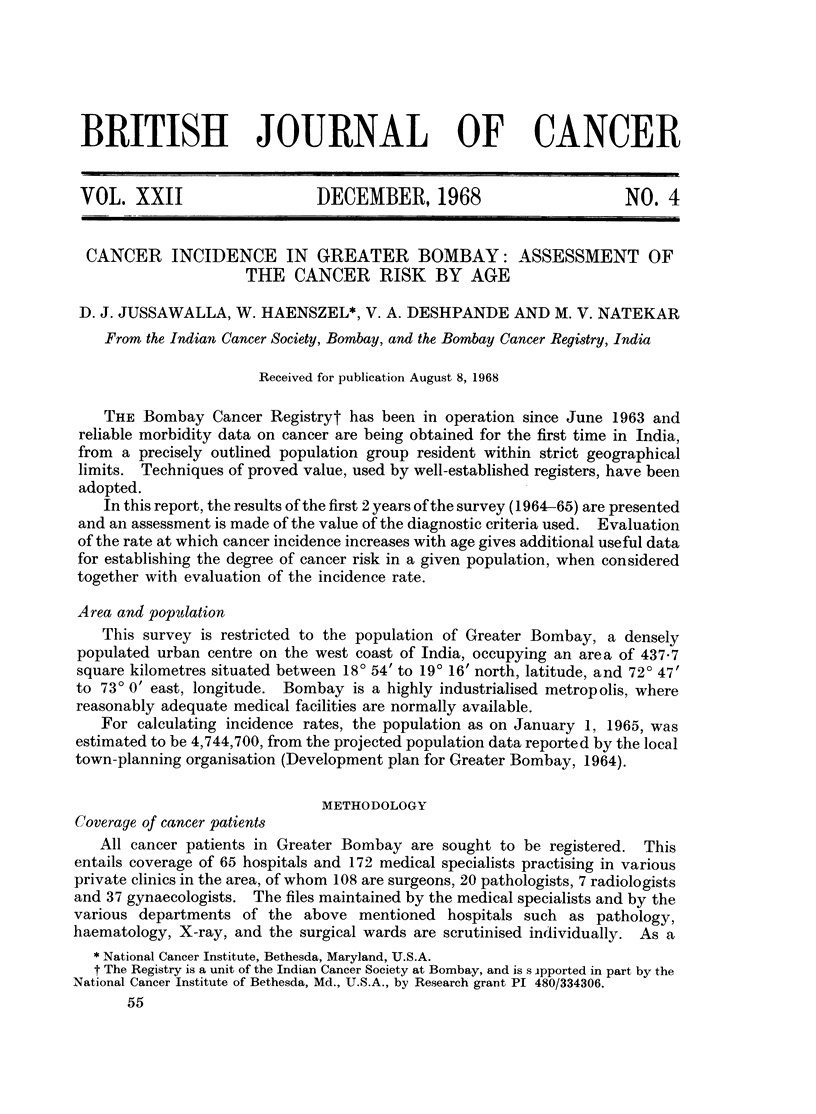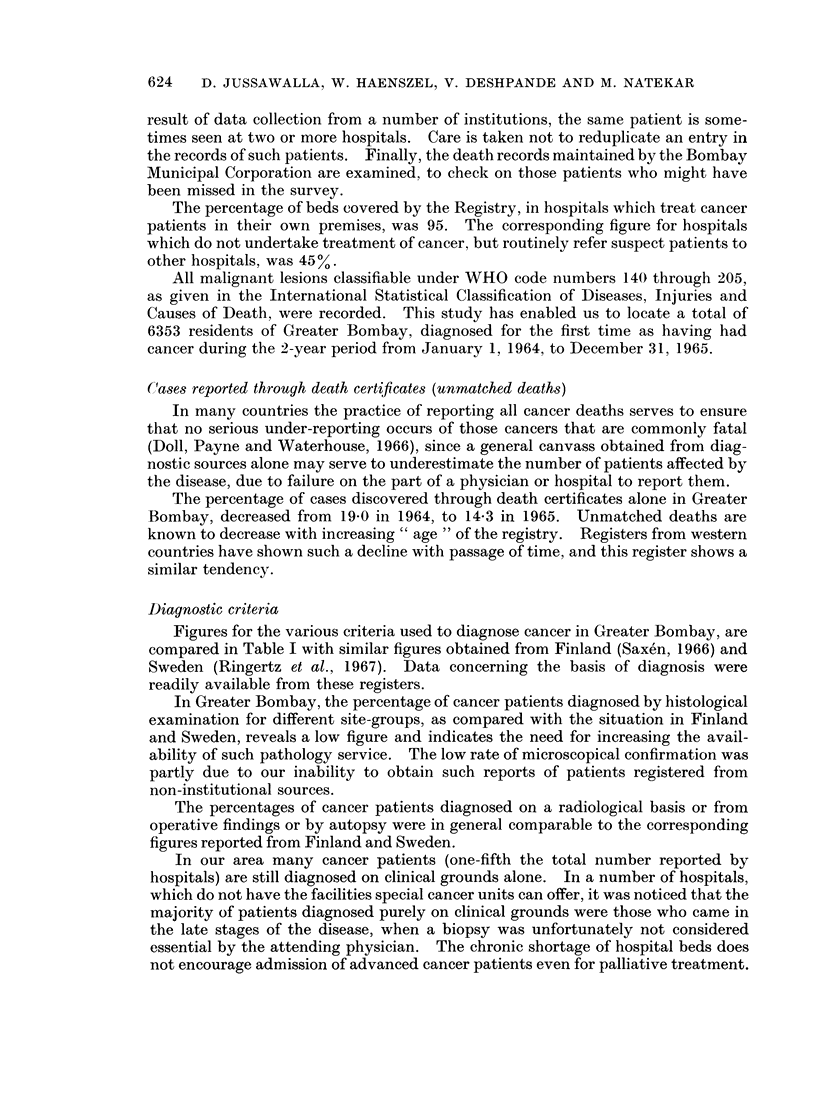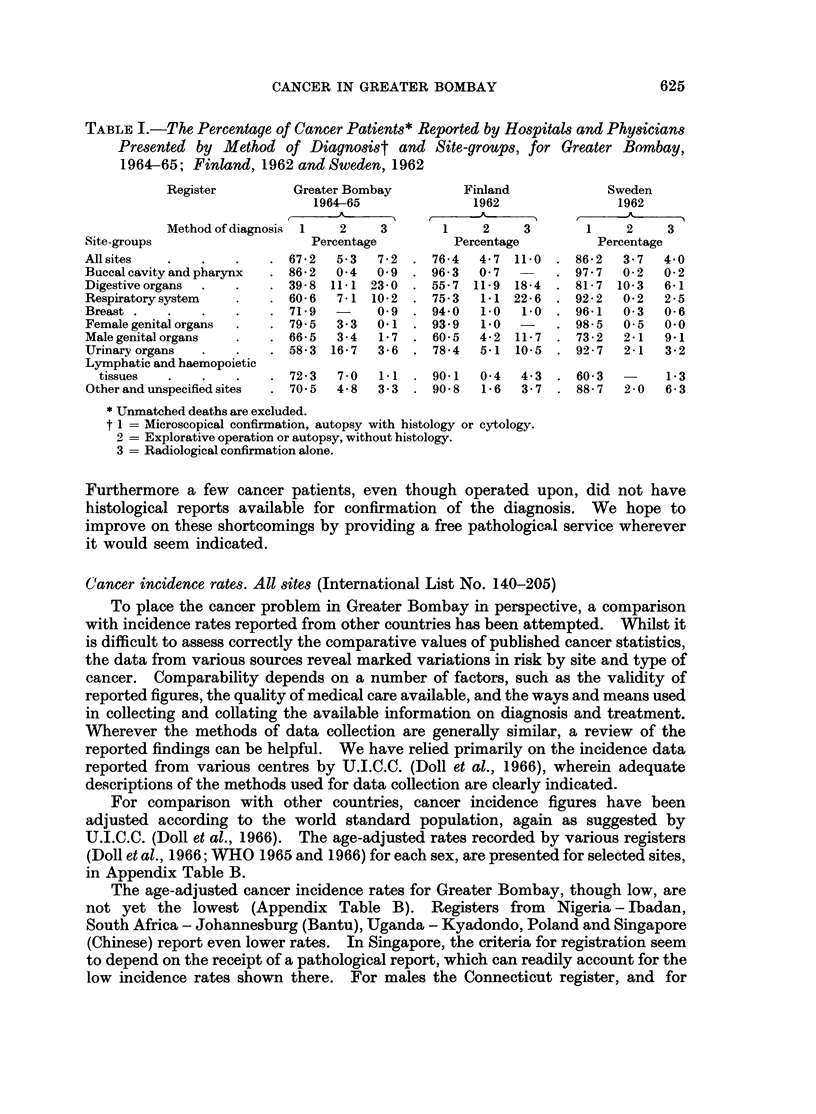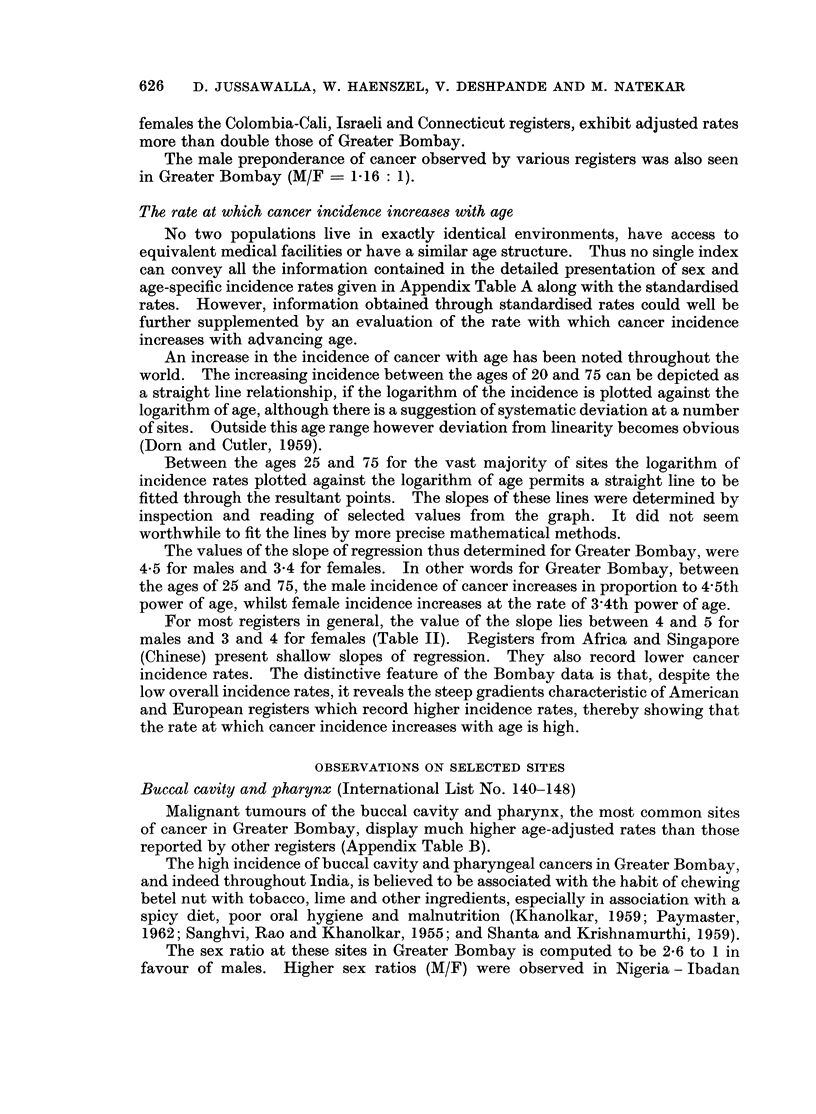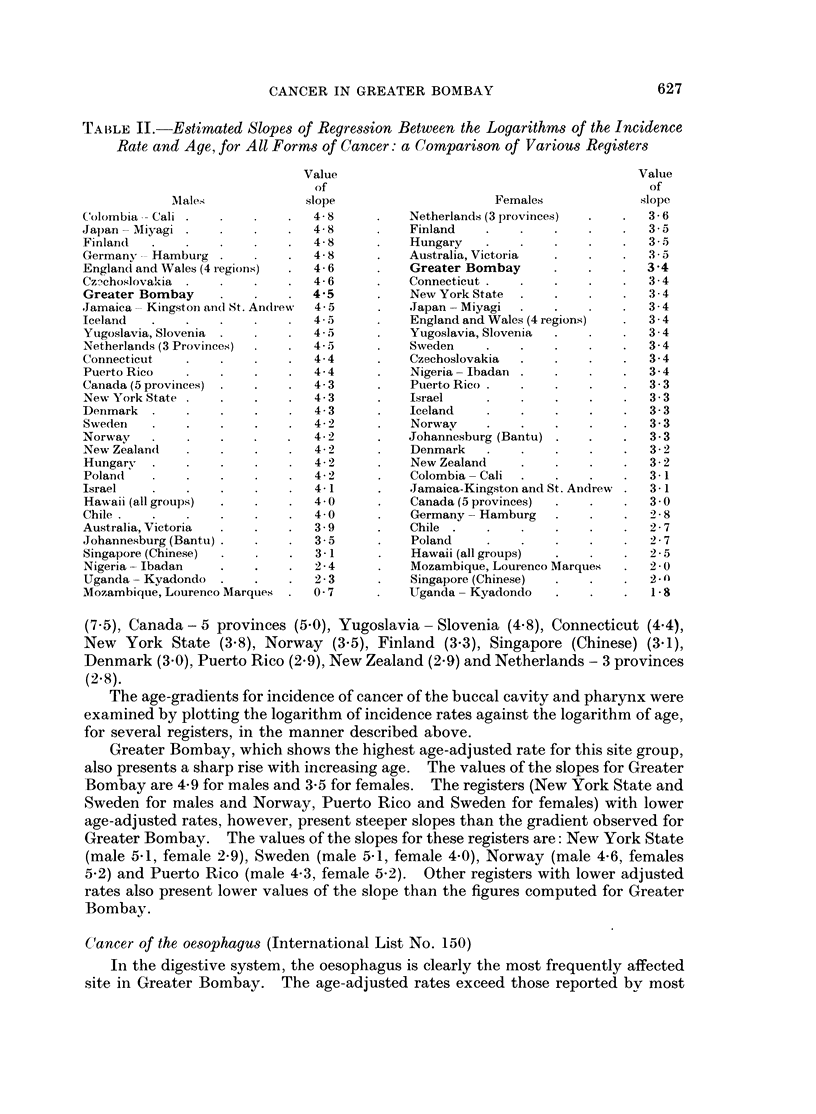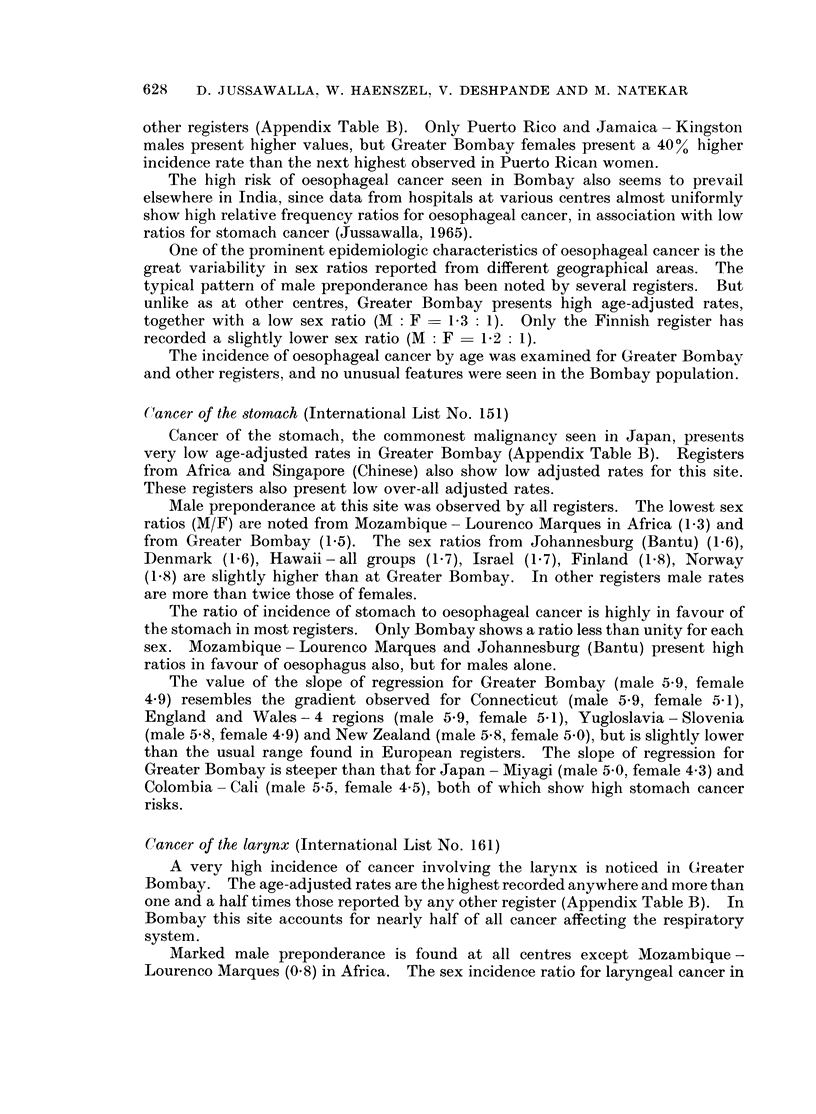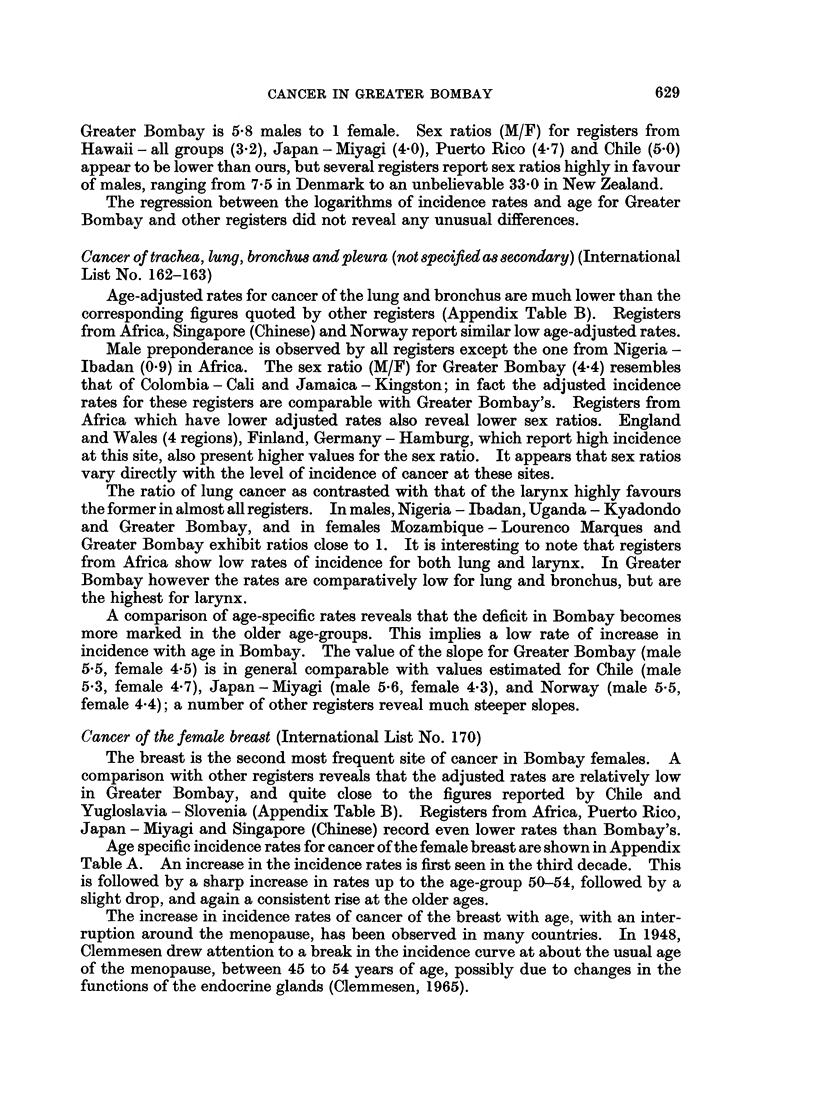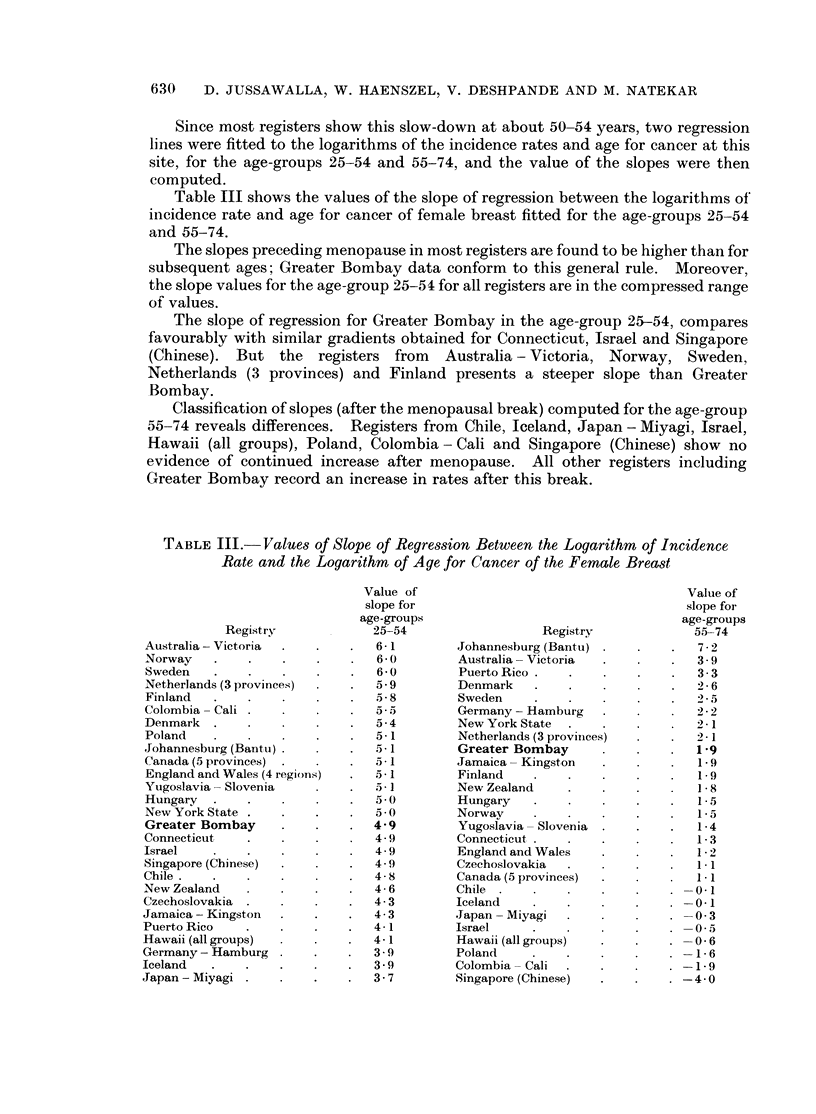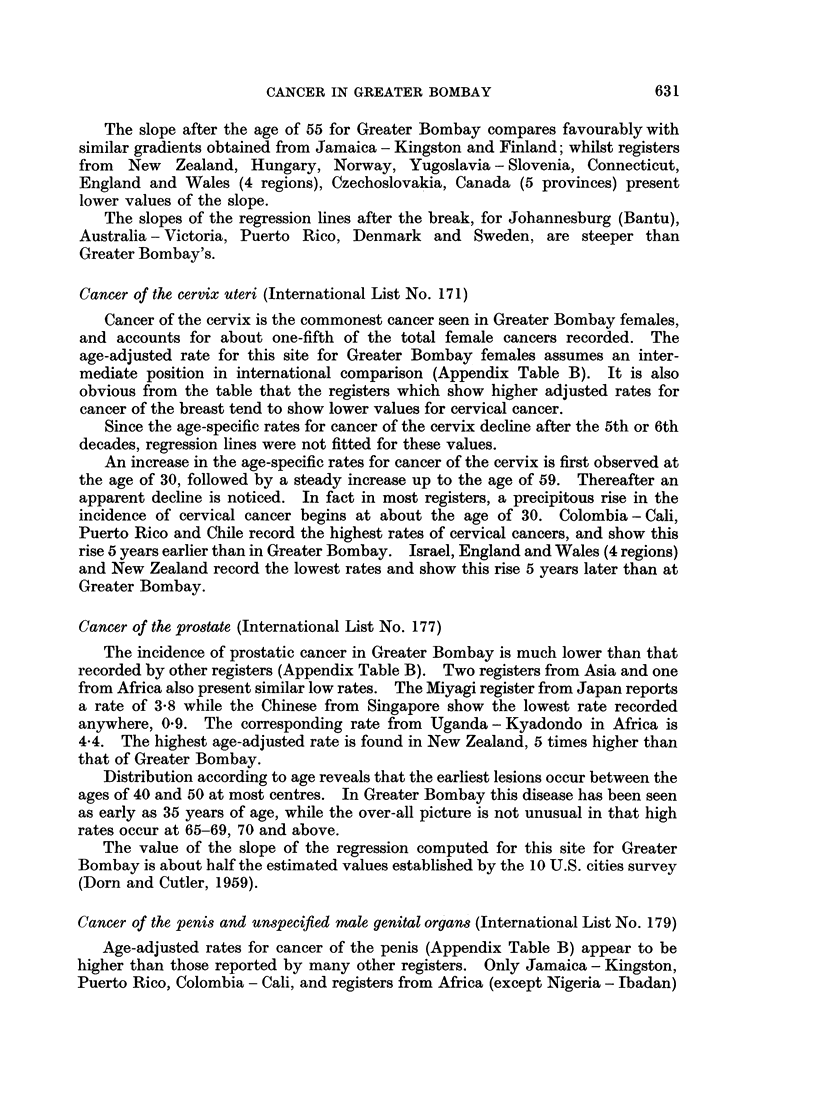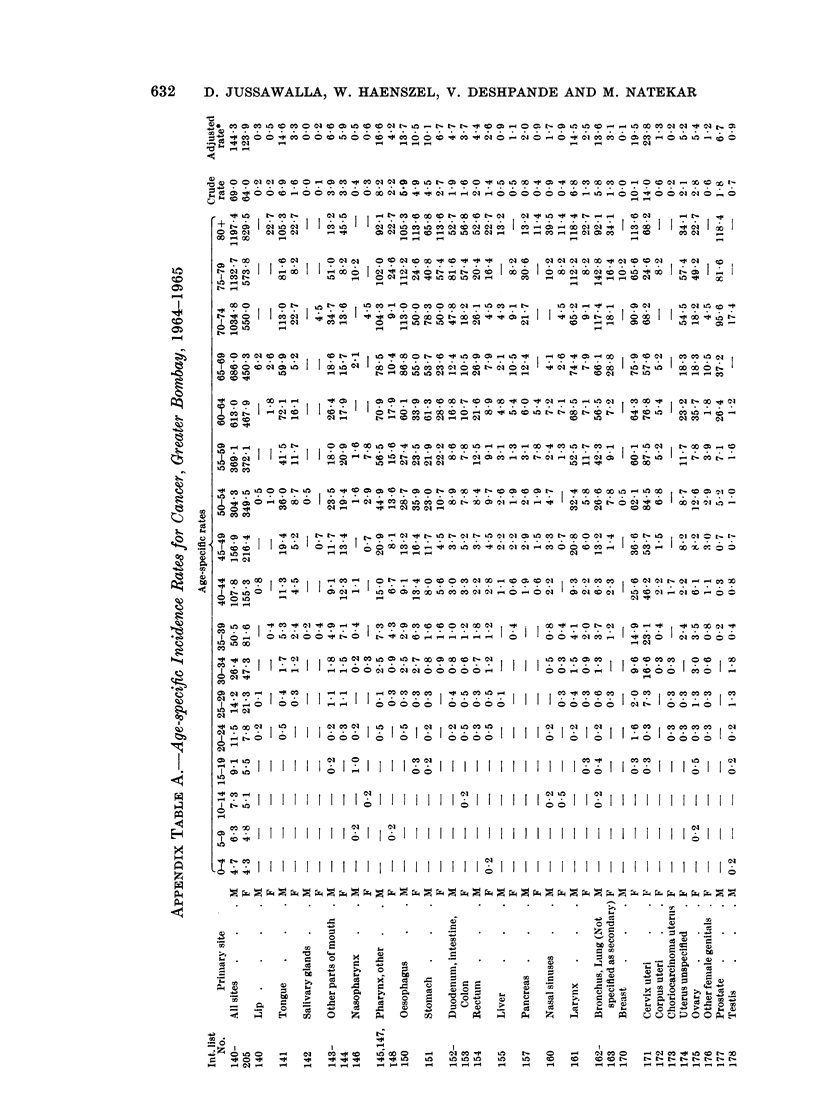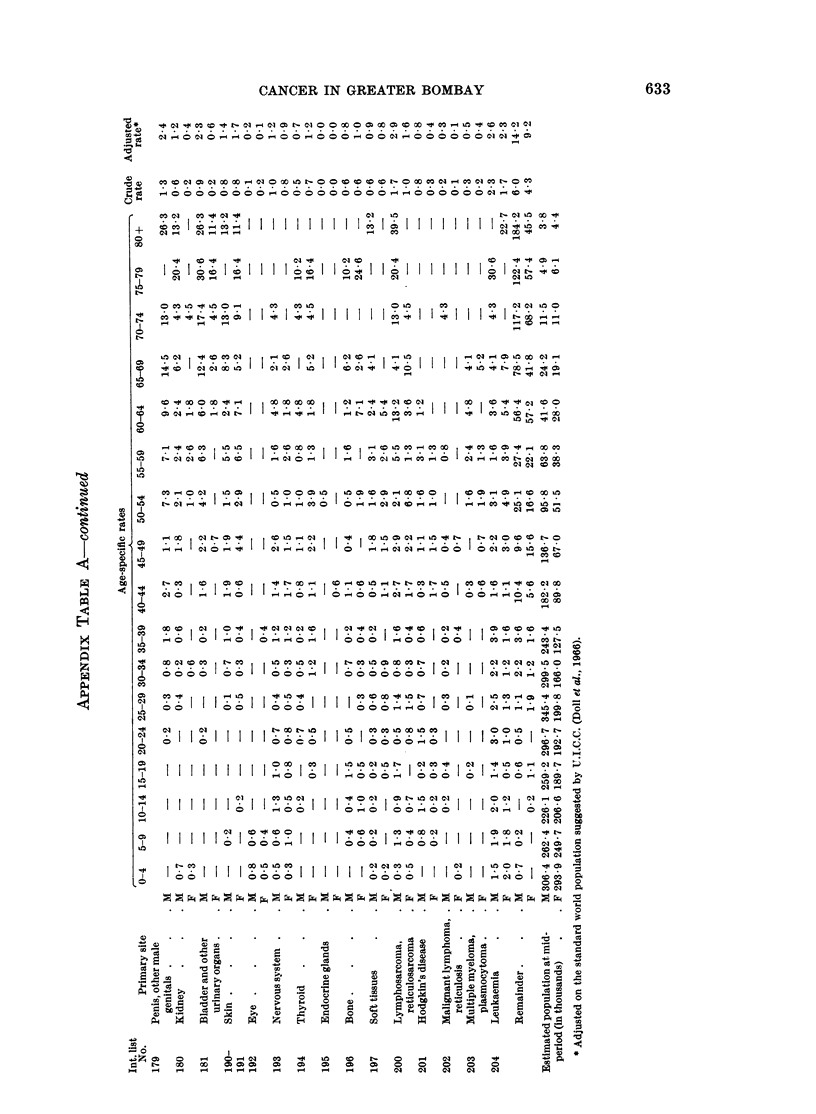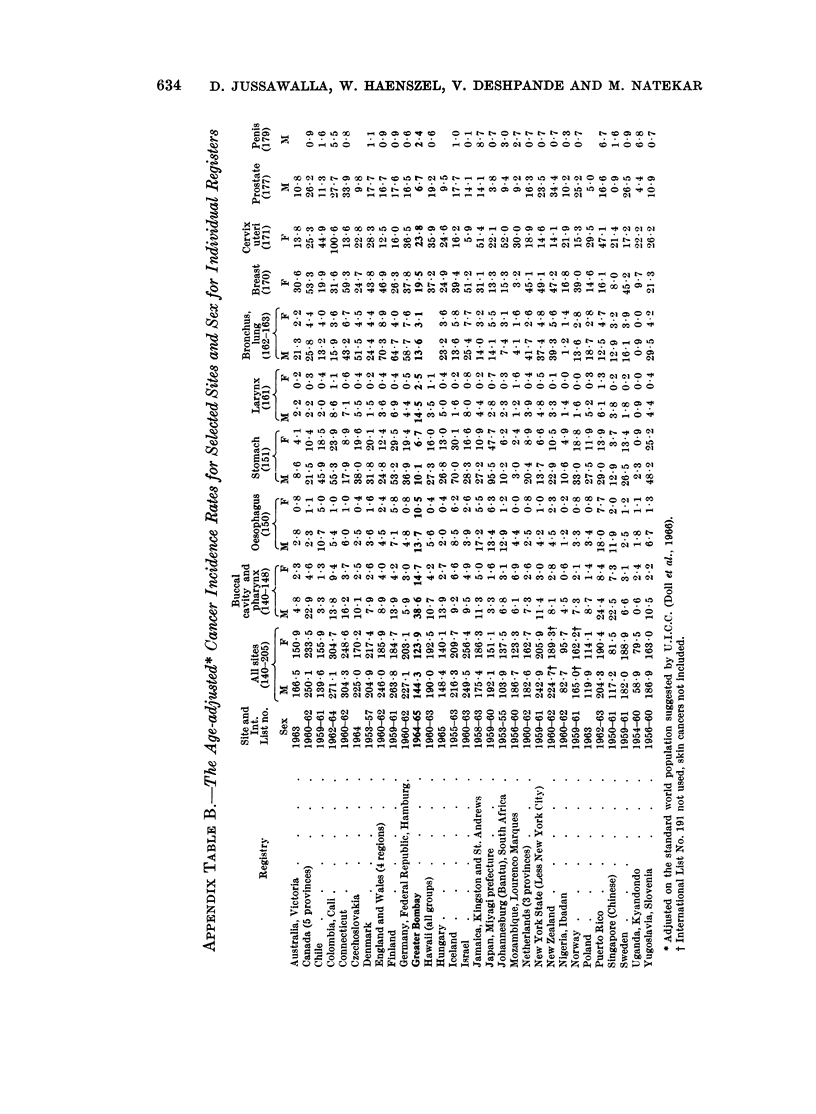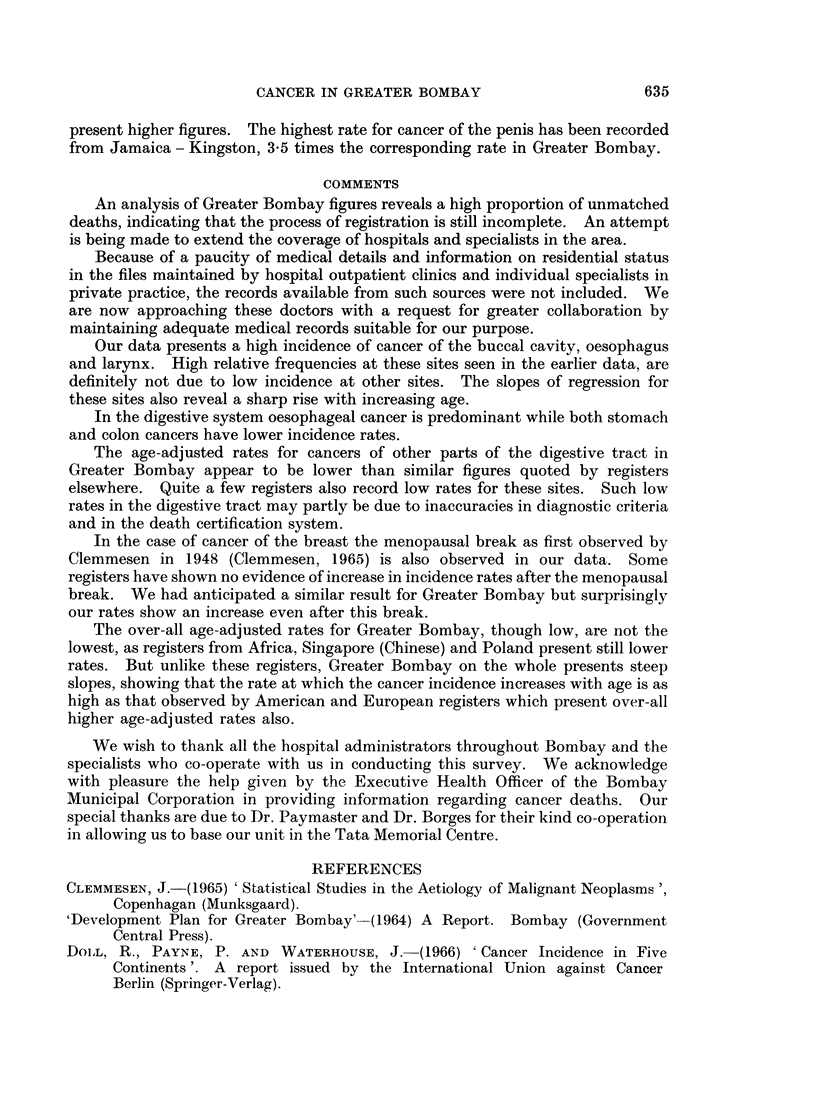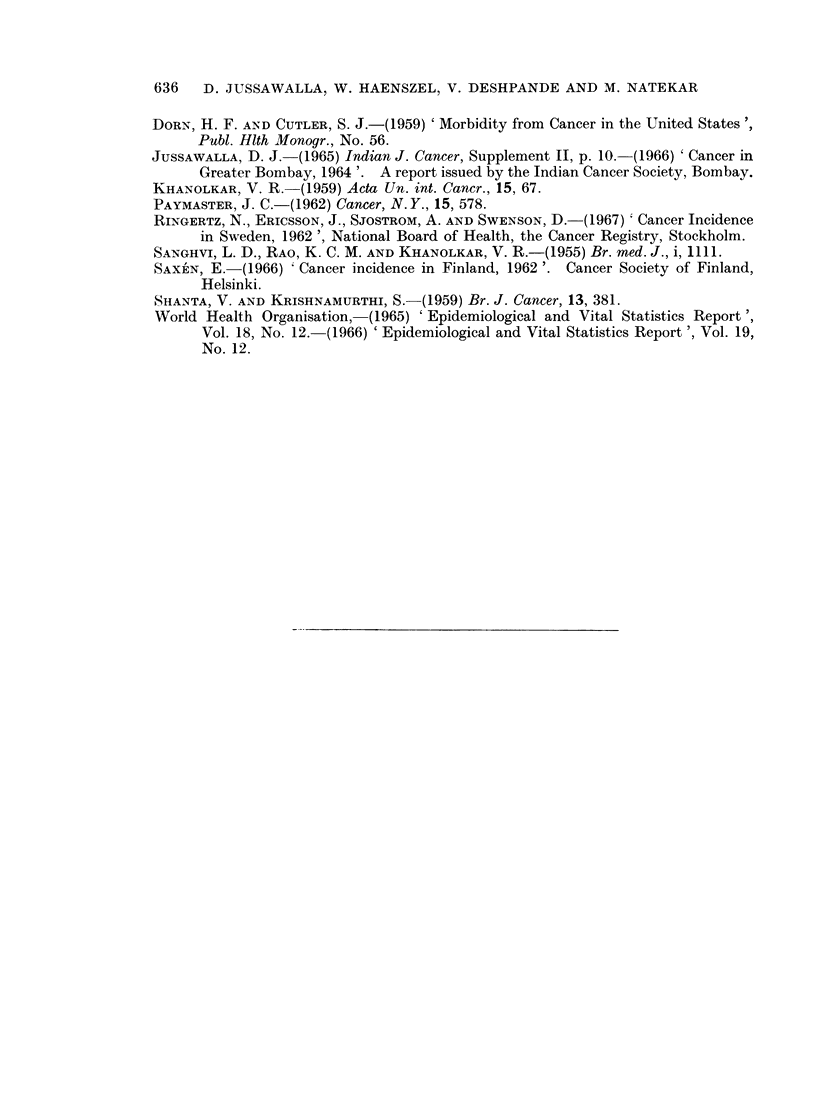# Cancer incidence in greater Bombay: assessment of the cancer risk by age.

**DOI:** 10.1038/bjc.1968.74

**Published:** 1968-12

**Authors:** D. J. Jussawalla, W. Haenszel, V. A. Deshpande, M. V. Natekar


BRITISH JOURNAL OF CANCER

VOL. XXII DECEMBER, 1968 NO. 4

### CANCER INCIDENCE IN GREATER BOMBAY: ASSESSMENT OF THE CANCER RISK BY AGE

D. J. JUSSAWALLA, W. HAENSZEL*, V. A. DESHPANDE AND M. V. NATEKAR

*From the Indian Cancer Society, Bombay, and the Bombay Cancer Registry, India*

Received for publication August 8, 1968

THE Bombay Cancer Registry† has been in operation since June 1963 and reliable morbidity data on cancer are being obtained for the first time in India, from a precisely outlined population group resident within strict geographical limits. Techniques of proved value, used by well-established registers, have been adopted.

In this report, the results of the first 2 years of the survey (1964–65) are presented and an assessment is made of the value of the diagnostic criteria used. Evaluation of the rate at which cancer incidence increases with age gives additional useful data for establishing the degree of cancer risk in a given population, when considered together with evaluation of the incidence rate.

*Area and population*

This survey is restricted to the population of Greater Bombay, a densely populated urban centre on the west coast of India, occupying an area of 437·7 square kilometres situated between 18° 54′ to 19° 16′ north, latitude, and 72° 47′ to 73° 0′ east, longitude. Bombay is a highly industrialised metropolis, where reasonably adequate medical facilities are normally available.

For calculating incidence rates, the population as on January 1, 1965, was estimated to be 4,744,700, from the projected population data reported by the local town-planning organisation (Development plan for Greater Bombay, 1964).

### METHODOLOGY

*Coverage of cancer patients*

All cancer patients in Greater Bombay are sought to be registered. This entails coverage of 65 hospitals and 172 medical specialists practising in various private clinics in the area, of whom 108 are surgeons, 20 pathologists, 7 radiologists and 37 gynaecologists. The files maintained by the medical specialists and by the various departments of the above mentioned hospitals such as pathology, haematology, X-ray, and the surgical wards are scrutinised individually. As a

* National Cancer Institute, Bethesda, Maryland, U.S.A.

† The Registry is a unit of the Indian Cancer Society at Bombay, and is supported in part by the National Cancer Institute of Bethesda, Md., U.S.A., by Research grant PI 480/334306.

result of data collection from a number of institutions, the same patient is sometimes seen at two or more hospitals. Care is taken not to reduplicate an entry in the records of such patients. Finally, the death records maintained by the Bombay Municipal Corporation are examined, to check on those patients who might have been missed in the survey.

The percentage of beds covered by the Registry, in hospitals which treat cancer patients in their own premises, was 95. The corresponding figure for hospitals which do not undertake treatment of cancer, but routinely refer suspect patients to other hospitals, was 45%.

All malignant lesions classifiable under WHO code numbers 140 through 205, as given in the International Statistical Classification of Diseases, Injuries and Causes of Death, were recorded. This study has enabled us to locate a total of 6353 residents of Greater Bombay, diagnosed for the first time as having had cancer during the 2-year period from January 1, 1964, to December 31, 1965.

*Cases reported through death certificates (unmatched deaths)*

In many countries the practice of reporting all cancer deaths serves to ensure that no serious under-reporting occurs of those cancers that are commonly fatal (Doll, Payne and Waterhouse, 1966), since a general canvass obtained from diagnostic sources alone may serve to underestimate the number of patients affected by the disease, due to failure on the part of a physician or hospital to report them.

The percentage of cases discovered through death certificates alone in Greater Bombay, decreased from 19·0 in 1964, to 14·3 in 1965. Unmatched deaths are known to decrease with increasing " age " of the registry. Registers from western countries have shown such a decline with passage of time, and this register shows a similar tendency.

*Diagnostic criteria*

Figures for the various criteria used to diagnose cancer in Greater Bombay, are compared in Table I with similar figures obtained from Finland (Saxén, 1966) and Sweden (Ringertz *et al.*, 1967). Data concerning the basis of diagnosis were readily available from these registers.

In Greater Bombay, the percentage of cancer patients diagnosed by histological examination for different site-groups, as compared with the situation in Finland and Sweden, reveals a low figure and indicates the need for increasing the availability of such pathology service. The low rate of microscopical confirmation was partly due to our inability to obtain such reports of patients registered from non-institutional sources.

The percentages of cancer patients diagnosed on a radiological basis or from operative findings or by autopsy were in general comparable to the corresponding figures reported from Finland and Sweden.

In our area many cancer patients (one-fifth the total number reported by hospitals) are still diagnosed on clinical grounds alone. In a number of hospitals, which do not have the facilities special cancer units can offer, it was noticed that the majority of patients diagnosed purely on clinical grounds were those who came in the late stages of the disease, when a biopsy was unfortunately not considered essential by the attending physician. The chronic shortage of hospital beds does not encourage admission of advanced cancer patients even for palliative treatment.

TABLE I.—*The Percentage of Cancer Patients* Reported by Hospitals and Physicians Presented by Method of Diagnosis† and Site-groups, for Greater Bombay,* 1964–65; *Finland,* 1962 *and Sweden,* 1962

| Register | Greater Bombay 1964–65 | | | Finland 1962 | | | Sweden 1962 | | |
|---|---|---|---|---|---|---|---|---|---|
| Method of diagnosis | 1 | 2 | 3 | 1 | 2 | 3 | 1 | 2 | 3 |
| Site-groups | Percentage | | | Percentage | | | Percentage | | |
| All sites | 67·2 | 5·3 | 7·2 | 76·4 | 4·7 | 11·0 | 86·2 | 3·7 | 4·0 |
| Buccal cavity and pharynx | 86·2 | 0·4 | 0·9 | 96·3 | 0·7 | — | 97·7 | 0·2 | 0·2 |
| Digestive organs | 39·8 | 11·1 | 23·0 | 55·7 | 11·9 | 18·4 | 81·7 | 10·3 | 6·1 |
| Respiratory system | 60·6 | 7·1 | 10·2 | 75·3 | 1·1 | 22·6 | 92·2 | 0·2 | 2·5 |
| Breast | 71·9 | — | 0·9 | 94·0 | 1·0 | 1·0 | 96·1 | 0·3 | 0·6 |
| Female genital organs | 79·5 | 3·3 | 0·1 | 93·9 | 1·0 | — | 98·5 | 0·5 | 0·0 |
| Male genital organs | 66·5 | 3·4 | 1·7 | 60·5 | 4·2 | 11·7 | 73·2 | 2·1 | 9·1 |
| Urinary organs | 58·3 | 16·7 | 3·6 | 78·4 | 5·1 | 10·5 | 92·7 | 2·1 | 3·2 |
| Lymphatic and haemopoietic tissues | 72·3 | 7·0 | 1·1 | 90·1 | 0·4 | 4·3 | 60·3 | — | 1·3 |
| Other and unspecified sites | 70·5 | 4·8 | 3·3 | 90·8 | 1·6 | 3·7 | 88·7 | 2·0 | 6·3 |

* Unmatched deaths are excluded.
† 1 = Microscopical confirmation, autopsy with histology or cytology.
2 = Explorative operation or autopsy, without histology.
3 = Radiological confirmation alone.

Furthermore a few cancer patients, even though operated upon, did not have histological reports available for confirmation of the diagnosis. We hope to improve on these shortcomings by providing a free pathological service wherever it would seem indicated.

*Cancer incidence rates. All sites* (International List No. 140–205)

To place the cancer problem in Greater Bombay in perspective, a comparison with incidence rates reported from other countries has been attempted. Whilst it is difficult to assess correctly the comparative values of published cancer statistics, the data from various sources reveal marked variations in risk by site and type of cancer. Comparability depends on a number of factors, such as the validity of reported figures, the quality of medical care available, and the ways and means used in collecting and collating the available information on diagnosis and treatment. Wherever the methods of data collection are generally similar, a review of the reported findings can be helpful. We have relied primarily on the incidence data reported from various centres by U.I.C.C. (Doll *et al.*, 1966), wherein adequate descriptions of the methods used for data collection are clearly indicated.

For comparison with other countries, cancer incidence figures have been adjusted according to the world standard population, again as suggested by U.I.C.C. (Doll *et al.*, 1966). The age-adjusted rates recorded by various registers (Doll *et al.*, 1966; WHO 1965 and 1966) for each sex, are presented for selected sites, in Appendix Table B.

The age-adjusted cancer incidence rates for Greater Bombay, though low, are not yet the lowest (Appendix Table B). Registers from Nigeria – Ibadan, South Africa – Johannesburg (Bantu), Uganda – Kyadondo, Poland and Singapore (Chinese) report even lower rates. In Singapore, the criteria for registration seem to depend on the receipt of a pathological report, which can readily account for the low incidence rates shown there. For males the Connecticut register, and for females the Colombia-Cali, Israeli and Connecticut registers, exhibit adjusted rates more than double those of Greater Bombay.

The male preponderance of cancer observed by various registers was also seen in Greater Bombay (M/F = 1·16 : 1).

*The rate at which cancer incidence increases with age*

No two populations live in exactly identical environments, have access to equivalent medical facilities or have a similar age structure. Thus no single index can convey all the information contained in the detailed presentation of sex and age-specific incidence rates given in Appendix Table A along with the standardised rates. However, information obtained through standardised rates could well be further supplemented by an evaluation of the rate with which cancer incidence increases with advancing age.

An increase in the incidence of cancer with age has been noted throughout the world. The increasing incidence between the ages of 20 and 75 can be depicted as a straight line relationship, if the logarithm of the incidence is plotted against the logarithm of age, although there is a suggestion of systematic deviation at a number of sites. Outside this age range however deviation from linearity becomes obvious (Dorn and Cutler, 1959).

Between the ages 25 and 75 for the vast majority of sites the logarithm of incidence rates plotted against the logarithm of age permits a straight line to be fitted through the resultant points. The slopes of these lines were determined by inspection and reading of selected values from the graph. It did not seem worthwhile to fit the lines by more precise mathematical methods.

The values of the slope of regression thus determined for Greater Bombay, were 4·5 for males and 3·4 for females. In other words for Greater Bombay, between the ages of 25 and 75, the male incidence of cancer increases in proportion to 4·5th power of age, whilst female incidence increases at the rate of 3·4th power of age.

For most registers in general, the value of the slope lies between 4 and 5 for males and 3 and 4 for females (Table II). Registers from Africa and Singapore (Chinese) present shallow slopes of regression. They also record lower cancer incidence rates. The distinctive feature of the Bombay data is that, despite the low overall incidence rates, it reveals the steep gradients characteristic of American and European registers which record higher incidence rates, thereby showing that the rate at which cancer incidence increases with age is high.

### OBSERVATIONS ON SELECTED SITES

*Buccal cavity and pharynx* (International List No. 140–148)

Malignant tumours of the buccal cavity and pharynx, the most common sites of cancer in Greater Bombay, display much higher age-adjusted rates than those reported by other registers (Appendix Table B).

The high incidence of buccal cavity and pharyngeal cancers in Greater Bombay, and indeed throughout India, is believed to be associated with the habit of chewing betel nut with tobacco, lime and other ingredients, especially in association with a spicy diet, poor oral hygiene and malnutrition (Khanolkar, 1959; Paymaster, 1962; Sanghvi, Rao and Khanolkar, 1955; and Shanta and Krishnamurthi, 1959).

The sex ratio at these sites in Greater Bombay is computed to be 2·6 to 1 in favour of males. Higher sex ratios (M/F) were observed in Nigeria – Ibadan

TABLE II.—*Estimated Slopes of Regression Between the Logarithms of the Incidence Rate and Age, for All Forms of Cancer: a Comparison of Various Registers*

| Males | Value of slope | Females | Value of slope |
|---|---|---|---|
| Colombia – Cali | 4·8 | Netherlands (3 provinces) | 3·6 |
| Japan – Miyagi | 4·8 | Finland | 3·5 |
| Finland | 4·8 | Hungary | 3·5 |
| Germany – Hamburg | 4·8 | Australia, Victoria | 3·5 |
| England and Wales (4 regions) | 4·6 | **Greater Bombay** | **3·4** |
| Czechoslovakia | 4·6 | Connecticut | 3·4 |
| **Greater Bombay** | **4·5** | New York State | 3·4 |
| Jamaica – Kingston and St. Andrew | 4·5 | Japan – Miyagi | 3·4 |
| Iceland | 4·5 | England and Wales (4 regions) | 3·4 |
| Yugoslavia, Slovenia | 4·5 | Yugoslavia, Slovenia | 3·4 |
| Netherlands (3 Provinces) | 4·5 | Sweden | 3·4 |
| Connecticut | 4·4 | Czechoslovakia | 3·4 |
| Puerto Rico | 4·4 | Nigeria – Ibadan | 3·4 |
| Canada (5 provinces) | 4·3 | Puerto Rico | 3·3 |
| New York State | 4·3 | Israel | 3·3 |
| Denmark | 4·3 | Iceland | 3·3 |
| Sweden | 4·2 | Norway | 3·3 |
| Norway | 4·2 | Johannesburg (Bantu) | 3·3 |
| New Zealand | 4·2 | Denmark | 3·2 |
| Hungary | 4·2 | New Zealand | 3·2 |
| Poland | 4·2 | Colombia – Cali | 3·1 |
| Israel | 4·1 | Jamaica-Kingston and St. Andrew | 3·1 |
| Hawaii (all groups) | 4·0 | Canada (5 provinces) | 3·0 |
| Chile | 4·0 | Germany – Hamburg | 2·8 |
| Australia, Victoria | 3·9 | Chile | 2·7 |
| Johannesburg (Bantu) | 3·5 | Poland | 2·7 |
| Singapore (Chinese) | 3·1 | Hawaii (all groups) | 2·5 |
| Nigeria – Ibadan | 2·4 | Mozambique, Lourenco Marques | 2·0 |
| Uganda – Kyadondo | 2·3 | Singapore (Chinese) | 2·0 |
| Mozambique, Lourenco Marques | 0·7 | Uganda – Kyadondo | 1·8 |

(7·5), Canada – 5 provinces (5·0), Yugoslavia – Slovenia (4·8), Connecticut (4·4), New York State (3·8), Norway (3·5), Finland (3·3), Singapore (Chinese) (3·1), Denmark (3·0), Puerto Rico (2·9), New Zealand (2·9) and Netherlands – 3 provinces (2·8).

The age-gradients for incidence of cancer of the buccal cavity and pharynx were examined by plotting the logarithm of incidence rates against the logarithm of age, for several registers, in the manner described above.

Greater Bombay, which shows the highest age-adjusted rate for this site group, also presents a sharp rise with increasing age. The values of the slopes for Greater Bombay are 4·9 for males and 3·5 for females. The registers (New York State and Sweden for males and Norway, Puerto Rico and Sweden for females) with lower age-adjusted rates, however, present steeper slopes than the gradient observed for Greater Bombay. The values of the slopes for these registers are: New York State (male 5·1, female 2·9), Sweden (male 5·1, female 4·0), Norway (male 4·6, females 5·2) and Puerto Rico (male 4·3, female 5·2). Other registers with lower adjusted rates also present lower values of the slope than the figures computed for Greater Bombay.

*Cancer of the oesophagus* (International List No. 150)

In the digestive system, the oesophagus is clearly the most frequently affected site in Greater Bombay. The age-adjusted rates exceed those reported by most other registers (Appendix Table B). Only Puerto Rico and Jamaica – Kingston males present higher values, but Greater Bombay females present a 40% higher incidence rate than the next highest observed in Puerto Rican women.

The high risk of oesophageal cancer seen in Bombay also seems to prevail elsewhere in India, since data from hospitals at various centres almost uniformly show high relative frequency ratios for oesophageal cancer, in association with low ratios for stomach cancer (Jussawalla, 1965).

One of the prominent epidemiologic characteristics of oesophageal cancer is the great variability in sex ratios reported from different geographical areas. The typical pattern of male preponderance has been noted by several registers. But unlike as at other centres, Greater Bombay presents high age-adjusted rates, together with a low sex ratio (M : F = 1·3 : 1). Only the Finnish register has recorded a slightly lower sex ratio (M : F = 1·2 : 1).

The incidence of oesophageal cancer by age was examined for Greater Bombay and other registers, and no unusual features were seen in the Bombay population.

*Cancer of the stomach* (International List No. 151)

Cancer of the stomach, the commonest malignancy seen in Japan, presents very low age-adjusted rates in Greater Bombay (Appendix Table B). Registers from Africa and Singapore (Chinese) also show low adjusted rates for this site. These registers also present low over-all adjusted rates.

Male preponderance at this site was observed by all registers. The lowest sex ratios (M/F) are noted from Mozambique – Lourenco Marques in Africa (1·3) and from Greater Bombay (1·5). The sex ratios from Johannesburg (Bantu) (1·6), Denmark (1·6), Hawaii – all groups (1·7), Israel (1·7), Finland (1·8), Norway (1·8) are slightly higher than at Greater Bombay. In other registers male rates are more than twice those of females.

The ratio of incidence of stomach to oesophageal cancer is highly in favour of the stomach in most registers. Only Bombay shows a ratio less than unity for each sex. Mozambique – Lourenco Marques and Johannesburg (Bantu) present high ratios in favour of oesophagus also, but for males alone.

The value of the slope of regression for Greater Bombay (male 5·9, female 4·9) resembles the gradient observed for Connecticut (male 5·9, female 5·1), England and Wales – 4 regions (male 5·9, female 5·1), Yugloslavia – Slovenia (male 5·8, female 4·9) and New Zealand (male 5·8, female 5·0), but is slightly lower than the usual range found in European registers. The slope of regression for Greater Bombay is steeper than that for Japan – Miyagi (male 5·0, female 4·3) and Colombia – Cali (male 5·5, female 4·5), both of which show high stomach cancer risks.

*Cancer of the larynx* (International List No. 161)

A very high incidence of cancer involving the larynx is noticed in Greater Bombay. The age-adjusted rates are the highest recorded anywhere and more than one and a half times those reported by any other register (Appendix Table B). In Bombay this site accounts for nearly half of all cancer affecting the respiratory system.

Marked male preponderance is found at all centres except Mozambique – Lourenco Marques (0·8) in Africa. The sex incidence ratio for laryngeal cancer in

Greater Bombay is 5·8 males to 1 female. Sex ratios (M/F) for registers from Hawaii – all groups (3·2), Japan – Miyagi (4·0), Puerto Rico (4·7) and Chile (5·0) appear to be lower than ours, but several registers report sex ratios highly in favour of males, ranging from 7·5 in Denmark to an unbelievable 33·0 in New Zealand.

The regression between the logarithms of incidence rates and age for Greater Bombay and other registers did not reveal any unusual differences.

*Cancer of trachea, lung, bronchus and pleura (not specified as secondary)* (International List No. 162–163)

Age-adjusted rates for cancer of the lung and bronchus are much lower than the corresponding figures quoted by other registers (Appendix Table B). Registers from Africa, Singapore (Chinese) and Norway report similar low age-adjusted rates.

Male preponderance is observed by all registers except the one from Nigeria – Ibadan (0·9) in Africa. The sex ratio (M/F) for Greater Bombay (4·4) resembles that of Colombia – Cali and Jamaica – Kingston; in fact the adjusted incidence rates for these registers are comparable with Greater Bombay's. Registers from Africa which have lower adjusted rates also reveal lower sex ratios. England and Wales (4 regions), Finland, Germany – Hamburg, which report high incidence at this site, also present higher values for the sex ratio. It appears that sex ratios vary directly with the level of incidence of cancer at these sites.

The ratio of lung cancer as contrasted with that of the larynx highly favours the former in almost all registers. In males, Nigeria – Ibadan, Uganda – Kyadondo and Greater Bombay, and in females Mozambique – Lourenco Marques and Greater Bombay exhibit ratios close to 1. It is interesting to note that registers from Africa show low rates of incidence for both lung and larynx. In Greater Bombay however the rates are comparatively low for lung and bronchus, but are the highest for larynx.

A comparison of age-specific rates reveals that the deficit in Bombay becomes more marked in the older age-groups. This implies a low rate of increase in incidence with age in Bombay. The value of the slope for Greater Bombay (male 5·5, female 4·5) is in general comparable with values estimated for Chile (male 5·3, female 4·7), Japan – Miyagi (male 5·6, female 4·3), and Norway (male 5·5, female 4·4); a number of other registers reveal much steeper slopes.

*Cancer of the female breast* (International List No. 170)

The breast is the second most frequent site of cancer in Bombay females. A comparison with other registers reveals that the adjusted rates are relatively low in Greater Bombay, and quite close to the figures reported by Chile and Yugloslavia – Slovenia (Appendix Table B). Registers from Africa, Puerto Rico, Japan – Miyagi and Singapore (Chinese) record even lower rates than Bombay's.

Age specific incidence rates for cancer of the female breast are shown in Appendix Table A. An increase in the incidence rates is first seen in the third decade. This is followed by a sharp increase in rates up to the age-group 50–54, followed by a slight drop, and again a consistent rise at the older ages.

The increase in incidence rates of cancer of the breast with age, with an interruption around the menopause, has been observed in many countries. In 1948, Clemmesen drew attention to a break in the incidence curve at about the usual age of the menopause, between 45 to 54 years of age, possibly due to changes in the functions of the endocrine glands (Clemmesen, 1965).

Since most registers show this slow-down at about 50–54 years, two regression lines were fitted to the logarithms of the incidence rates and age for cancer at this site, for the age-groups 25–54 and 55–74, and the value of the slopes were then computed.

Table III shows the values of the slope of regression between the logarithms of incidence rate and age for cancer of female breast fitted for the age-groups 25–54 and 55–74.

The slopes preceding menopause in most registers are found to be higher than for subsequent ages; Greater Bombay data conform to this general rule. Moreover, the slope values for the age-group 25–54 for all registers are in the compressed range of values.

The slope of regression for Greater Bombay in the age-group 25–54, compares favourably with similar gradients obtained for Connecticut, Israel and Singapore (Chinese). But the registers from Australia – Victoria, Norway, Sweden, Netherlands (3 provinces) and Finland presents a steeper slope than Greater Bombay.

Classification of slopes (after the menopausal break) computed for the age-group 55–74 reveals differences. Registers from Chile, Iceland, Japan – Miyagi, Israel, Hawaii (all groups), Poland, Colombia – Cali and Singapore (Chinese) show no evidence of continued increase after menopause. All other registers including Greater Bombay record an increase in rates after this break.

TABLE III.—*Values of Slope of Regression Between the Logarithm of Incidence Rate and the Logarithm of Age for Cancer of the Female Breast*

| Registry | Value of slope for age-groups 25–54 | Registry | Value of slope for age-groups 55–74 |
|---|---|---|---|
| Australia – Victoria | 6·1 | Johannesburg (Bantu) | 7·2 |
| Norway | 6·0 | Australia – Victoria | 3·9 |
| Sweden | 6·0 | Puerto Rico | 3·3 |
| Netherlands (3 provinces) | 5·9 | Denmark | 2·6 |
| Finland | 5·8 | Sweden | 2·5 |
| Colombia – Cali | 5·5 | Germany – Hamburg | 2·2 |
| Denmark | 5·4 | New York State | 2·1 |
| Poland | 5·1 | Netherlands (3 provinces) | 2·1 |
| Johannesburg (Bantu) | 5·1 | **Greater Bombay** | **1·9** |
| Canada (5 provinces) | 5·1 | Jamaica – Kingston | 1·9 |
| England and Wales (4 regions) | 5·1 | Finland | 1·9 |
| Yugoslavia – Slovenia | 5·1 | New Zealand | 1·8 |
| Hungary | 5·0 | Hungary | 1·5 |
| New York State | 5·0 | Norway | 1·5 |
| **Greater Bombay** | **4·9** | Yugoslavia – Slovenia | 1·4 |
| Connecticut | 4·9 | Connecticut | 1·3 |
| Israel | 4·9 | England and Wales | 1·2 |
| Singapore (Chinese) | 4·9 | Czechoslovakia | 1·1 |
| Chile | 4·8 | Canada (5 provinces) | 1·1 |
| New Zealand | 4·6 | Chile | −0·1 |
| Czechoslovakia | 4·3 | Iceland | −0·1 |
| Jamaica – Kingston | 4·3 | Japan – Miyagi | −0·3 |
| Puerto Rico | 4·1 | Israel | −0·5 |
| Hawaii (all groups) | 4·1 | Hawaii (all groups) | −0·6 |
| Germany – Hamburg | 3·9 | Poland | −1·6 |
| Iceland | 3·9 | Colombia – Cali | −1·9 |
| Japan – Miyagi | 3·7 | Singapore (Chinese) | −4·0 |

The slope after the age of 55 for Greater Bombay compares favourably with similar gradients obtained from Jamaica – Kingston and Finland; whilst registers from New Zealand, Hungary, Norway, Yugoslavia – Slovenia, Connecticut, England and Wales (4 regions), Czechoslovakia, Canada (5 provinces) present lower values of the slope.

The slopes of the regression lines after the break, for Johannesburg (Bantu), Australia – Victoria, Puerto Rico, Denmark and Sweden, are steeper than Greater Bombay's.

*Cancer of the cervix uteri* (International List No. 171)

Cancer of the cervix is the commonest cancer seen in Greater Bombay females, and accounts for about one-fifth of the total female cancers recorded. The age-adjusted rate for this site for Greater Bombay females assumes an intermediate position in international comparison (Appendix Table B). It is also obvious from the table that the registers which show higher adjusted rates for cancer of the breast tend to show lower values for cervical cancer.

Since the age-specific rates for cancer of the cervix decline after the 5th or 6th decades, regression lines were not fitted for these values.

An increase in the age-specific rates for cancer of the cervix is first observed at the age of 30, followed by a steady increase up to the age of 59. Thereafter an apparent decline is noticed. In fact in most registers, a precipitous rise in the incidence of cervical cancer begins at about the age of 30. Colombia – Cali, Puerto Rico and Chile record the highest rates of cervical cancers, and show this rise 5 years earlier than in Greater Bombay. Israel, England and Wales (4 regions) and New Zealand record the lowest rates and show this rise 5 years later than at Greater Bombay.

*Cancer of the prostate* (International List No. 177)

The incidence of prostatic cancer in Greater Bombay is much lower than that recorded by other registers (Appendix Table B). Two registers from Asia and one from Africa also present similar low rates. The Miyagi register from Japan reports a rate of 3·8 while the Chinese from Singapore show the lowest rate recorded anywhere, 0·9. The corresponding rate from Uganda – Kyadondo in Africa is 4·4. The highest age-adjusted rate is found in New Zealand, 5 times higher than that of Greater Bombay.

Distribution according to age reveals that the earliest lesions occur between the ages of 40 and 50 at most centres. In Greater Bombay this disease has been seen as early as 35 years of age, while the over-all picture is not unusual in that high rates occur at 65–69, 70 and above.

The value of the slope of the regression computed for this site for Greater Bombay is about half the estimated values established by the 10 U.S. cities survey (Dorn and Cutler, 1959).

*Cancer of the penis and unspecified male genital organs* (International List No. 179)

Age-adjusted rates for cancer of the penis (Appendix Table B) appear to be higher than those reported by many other registers. Only Jamaica – Kingston, Puerto Rico, Colombia – Cali, and registers from Africa (except Nigeria – Ibadan)

present higher figures. The highest rate for cancer of the penis has been recorded from Jamaica – Kingston, 3·5 times the corresponding rate in Greater Bombay.

COMMENTS

An analysis of Greater Bombay figures reveals a high proportion of unmatched deaths, indicating that the process of registration is still incomplete. An attempt is being made to extend the coverage of hospitals and specialists in the area.

Because of a paucity of medical details and information on residential status in the files maintained by hospital outpatient clinics and individual specialists in private practice, the records available from such sources were not included. We are now approaching these doctors with a request for greater collaboration by maintaining adequate medical records suitable for our purpose.

Our data presents a high incidence of cancer of the buccal cavity, oesophagus and larynx. High relative frequencies at these sites seen in the earlier data, are definitely not due to low incidence at other sites. The slopes of regression for these sites also reveal a sharp rise with increasing age.

In the digestive system oesophageal cancer is predominant while both stomach and colon cancers have lower incidence rates.

The age-adjusted rates for cancers of other parts of the digestive tract in Greater Bombay appear to be lower than similar figures quoted by registers elsewhere. Quite a few registers also record low rates for these sites. Such low rates in the digestive tract may partly be due to inaccuracies in diagnostic criteria and in the death certification system.

In the case of cancer of the breast the menopausal break as first observed by Clemmesen in 1948 (Clemmesen, 1965) is also observed in our data. Some registers have shown no evidence of increase in incidence rates after the menopausal break. We had anticipated a similar result for Greater Bombay but surprisingly our rates show an increase even after this break.

The over-all age-adjusted rates for Greater Bombay, though low, are not the lowest, as registers from Africa, Singapore (Chinese) and Poland present still lower rates. But unlike these registers, Greater Bombay on the whole presents steep slopes, showing that the rate at which the cancer incidence increases with age is as high as that observed by American and European registers which present over-all higher age-adjusted rates also.

We wish to thank all the hospital administrators throughout Bombay and the specialists who co-operate with us in conducting this survey. We acknowledge with pleasure the help given by the Executive Health Officer of the Bombay Municipal Corporation in providing information regarding cancer deaths. Our special thanks are due to Dr. Paymaster and Dr. Borges for their kind co-operation in allowing us to base our unit in the Tata Memorial Centre.

REFERENCES

CLEMMESEN, J.—(1965) ' Statistical Studies in the Aetiology of Malignant Neoplasms ', Copenhagan (Munksgaard).

'Development Plan for Greater Bombay'—(1964) A Report. Bombay (Government Central Press).

DOLL, R., PAYNE, P. AND WATERHOUSE, J.—(1966) ' Cancer Incidence in Five Continents '. A report issued by the International Union against Cancer Berlin (Springer-Verlag).

DORN, H. F. AND CUTLER, S. J.—(1959) ' Morbidity from Cancer in the United States ', *Publ. Hlth Monogr.*, No. 56.
JUSSAWALLA, D. J.—(1965) *Indian J. Cancer*, Supplement II, p. 10.—(1966) ' Cancer in Greater Bombay, 1964 '. A report issued by the Indian Cancer Society, Bombay.
KHANOLKAR, V. R.—(1959) *Acta Un. int. Cancr.*, **15**, 67.
PAYMASTER, J. C.—(1962) *Cancer, N.Y.*, **15**, 578.
RINGERTZ, N., ERICSSON, J., SJOSTROM, A. AND SWENSON, D.—(1967) ' Cancer Incidence in Sweden, 1962 ', National Board of Health, the Cancer Registry, Stockholm.
SANGHVI, L. D., RAO, K. C. M. AND KHANOLKAR, V. R.—(1955) *Br. med. J.*, i, 1111.
SAXÉN, E.—(1966) ' Cancer incidence in Finland, 1962 '. Cancer Society of Finland, Helsinki.
SHANTA, V. AND KRISHNAMURTHI, S.—(1959) *Br. J. Cancer*, **13**, 381.
World Health Organisation,—(1965) ' Epidemiological and Vital Statistics Report ', Vol. 18, No. 12.—(1966) ' Epidemiological and Vital Statistics Report ', Vol. 19, No. 12.